# Health and Social Determinants Associated With Delay of Health Care Among Rural Older Adults

**DOI:** 10.1093/geroni/igab046.813

**Published:** 2021-12-17

**Authors:** Autumn Decker, Raven Weaver

**Affiliations:** Washington State University, Pullman, Washington, United States

## Abstract

Delaying healthcare has the capacity to increase morbidity and mortality, especially among individuals with chronic and acute health conditions. Older adults in rural areas are more likely to have chronic health conditions and are more likely to delay care due to financial barriers than their urban counterparts. To further investigate these associations, we conducted descriptive, bivariate, and regression analyses using data from a needs assessment designed to identify health needs and service delivery gaps among an economically diverse eight-county region. A random sample of adults responded to the survey, with 1,226 respondents aged 60+ (mean age = 71). The majority of respondents were White, female, and had insurance coverage. Overall, 35% of respondents experienced a delay in healthcare. We used logistic regression to determine the associations of age, gender, number of health conditions, household income, distance from medical facility, and perceived quality of neighborhood with delay of healthcare. Individuals with younger age (p = .017), more chronic conditions (p < .001), lower income (p < .001), and lower perceived quality of neighborhood (p = .008) were more likely to experience a delay in healthcare. These findings highlight risk factors that were salient prior to the onset of the COVID-19 pandemic. However, the pandemic has contributed to an increasing trend of delaying healthcare and may have amplified existing challenges. Findings may inform efforts led by healthcare providers and policy makers to facilitate timely and preventive healthcare use. Future research is needed to investigate the compounding long-term health implications of delaying healthcare.

